# Platelet Power: Revitalizing Endodontics With Scaffolds

**DOI:** 10.7759/cureus.60691

**Published:** 2024-05-20

**Authors:** Palak Hirani, Manoj Chandak, Paridhi Agrawal, Swayangprabha Sarangi, Tejas Suryawanshi, Namrata Jidewar, Kapil Naladkar

**Affiliations:** 1 Department of Conservative Dentistry and Endodontics, Sharad Pawar Dental College and Hospital, Datta Meghe Institute of Higher Education and Research, Wardha, IND

**Keywords:** regenerative endodontics, platelet-rich plasma/ prp, prf membrane, platelet concentrates, scaffolds

## Abstract

This article provides an overview of a biologically based method for restoring damaged tooth structures and pulp tissues known as regenerative endodontics. It explores the concept of regenerative endodontics, its tissue engineering approach, and its application in maintaining vitality. The article discusses the significance of the factors affecting growth, scaffolds, and stem cells being the three tissue engineering components involved in the regeneration of pulp tissues. It also delves into the classification of scaffolds and the role of platelet-rich fibrin (PRF) and platelet-rich plasma (PRP) as biological scaffolds. The methodology section details the search process for relevant studies, and the review section presents research findings associated with PRF and its application in regeneration and repair of tissue. The article concludes by highlighting the potential of advanced PRF and injectable PRF in regenerative endodontics, with a focus on their impact on tissue regeneration and healing.

## Introduction and background

**Figure 1:**
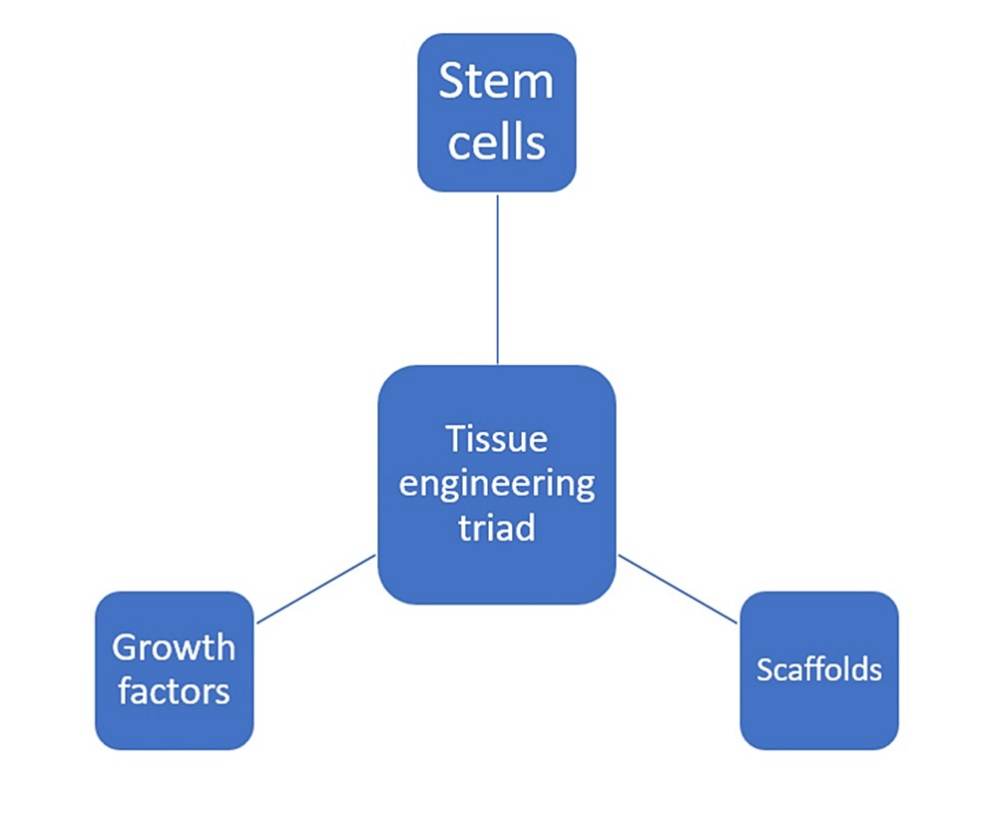
Regenerative Endodontics Triad Image Credits - Palak Hirani

"Biologically based procedures designed to replace damaged structures, including dentin and root structures, as well as cells of the pulp-dentin complex," is endodontic regeneration [1]. Based on a tissue engineering approach, the American Association of Endodontists accepted the term "regenerative endodontics" in 2007. Regenerative endodontics uses the tissue engineering triad - stem cells, scaffolds, and bioactive growth factors - to repair pulp tissues that have been harmed by infection, trauma, or developmental abnormalities [1]. Its goal is to maintain the teeth's sensitivity and vitality. Root maturation is the primary clinical advantage [2]. Regenerative endodontics has become a viable alternative for treating teeth that are non-vital. Rather than actual pulp dentin, undifferentiated cells are used to create new pulp tissue. Revascularization, also known as regenerative endodontic therapy (RET), has also been suggested as a treatment for these immature permanent teeth. Through the replacement of damaged root tissues and cells involved in radicular morphogenesis, RET seeks to both restore the blood supply and permit further root development [3]. This led to the development of "revascularization" in the cleaned canal, which is based on cell-homing, in the 2000s [4]. The tissue engineering triad comprises three distinct biological components, as seen in Figure 1: stem cells, scaffolds, and growth factors.

In the realm of regenerative treatments, the idea that stem cells, which are undifferentiated cells, can differentiate into a particular tissue on a scaffold or suitable media under the influence of growth factors or bioactive substances, is extremely plausible. The field of endodontic regeneration is a recent addition to the medical landscape. In cases of significant periapical lesions and endo-perio lesions, it deals with the bone, cementum, and periodontal ligament regeneration; in necrotic immature permanent teeth, it rejuvenates the pulp-dentin complex [5]. Scaffolds can be classified according to their form, size, degradability, origin, and presence or absence of cells. One such autologous and biological scaffold, the qualities of which are examined in this paper, is platelet concentrate.

## Review

Methodology

We searched the MEDLINE via PUBMED and Cochrane Central Register of Controlled Trials (CENTRAL) database through the Cochrane library for this narrative review. Several terms were used, “scaffolds”, “platelet concentrates”, “PRF”, “PRP”, and “Regenerative Endodontics”. To find any more research, we also looked through the reference lists of possible relevant studies. Studies retrieved from these computerized searches as well as pertinent references located in the study bibliographies were included in our review.

Stem cells

Cell homing and cell-based transplantation therapy are the two techniques that can be employed to restore the pulp-dentin complex. Intravenous or in situ transplantation of autologous or allogeneic stem/progenitor cells is the primary therapeutic approach [6,7]. Dental stem cells are thought to originate from populations of mesenchymal stem cells. Dental pulp stem cells (DPSC), apical papilla stem cells (SCAP), periodontal ligament stem cells, and human exfoliated deciduous tooth stem cells [8]. Because DPSC are multipotent cells that may differentiate into a variety of cell types, including osteoblasts, adipocytes, and neural cells, they have a great deal of promise for endodontic regeneration [9].

Growth factors

Polypeptides or proteins known as growth factors, trigger a range of cellular functions when bound, such as migration, proliferation, differentiation, and maturation [10,11]. Growth factors play a very important role in tissue regeneration and repair by modulating particular signalling pathways. Growth factors for cell homing might arise spontaneously from blood input, residual pulp components, stem cells, or neighbouring dentin. Recent research, however, has shown that regeneration can happen without the use of any exogenous growth factors [12]. Following disinfection, growth factors of endogenous origin which are located in the dentin wall may be released, enabling their utilization in the process of regeneration [13]. It has also been discovered that dentin is abundant in angiogenic growth factors [14].

Scaffolds

A scaffold is an intricate substance whose chemical and mechanical characteristics are similar to those of the extracellular matrix seen in nature [15]. Another way to describe it is as a three-dimensional microstructural network of biologically active substances that cooperate to guarantee the safe distribution of bioactive cells, which are crucial for promoting tissue regeneration and repair [5]. An effective scaffold should provide the necessary structural support for invading cells, as well as encourage their survival, division, and adherence as well as the extracellular matrix's deposition. The volume, shape, and size of the scaffold pores are critical for the removal of waste materials as well as the transportation of nutrients, growth factors (GF), and oxygen. Like with all biomaterials, the material must be both sufficiently strong mechanically and physiologically. Consequently, the scaffold needs to degrade at a pace that is appropriate for tissue regeneration in order for the tissue to fully regenerate [16].

Ideal Requirements

**Figure 2:**
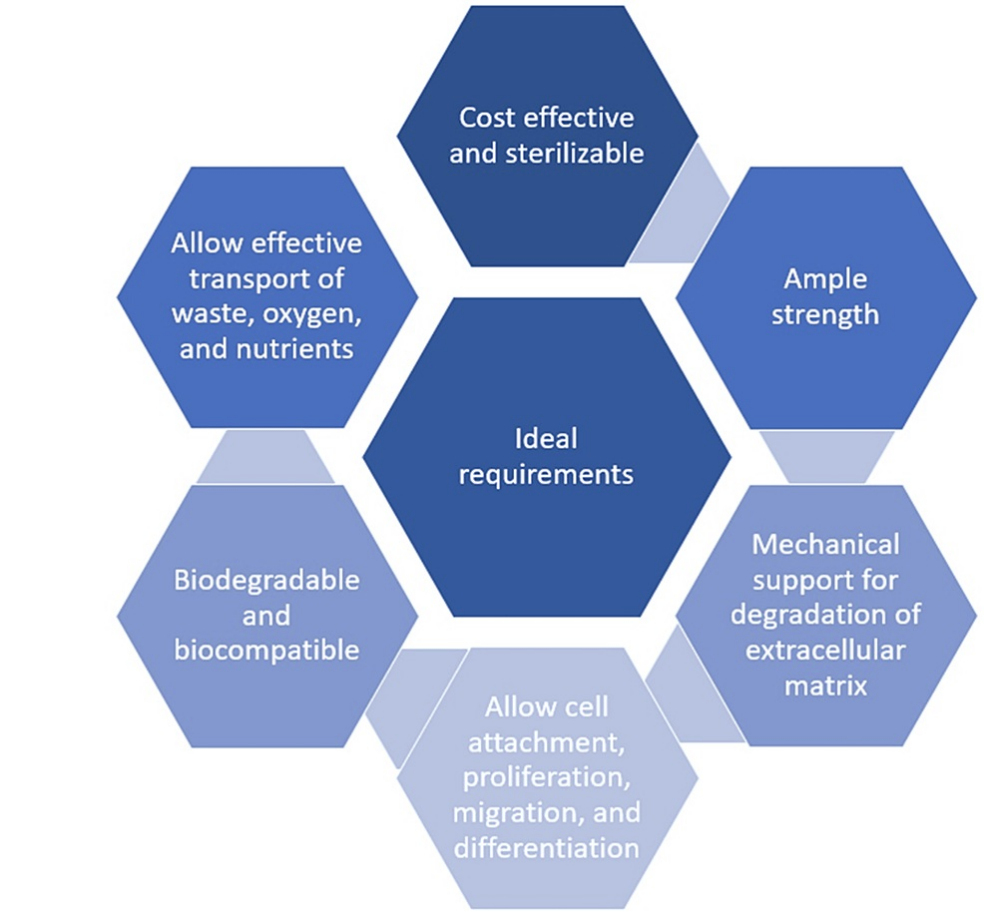
Ideal Requirements Of Scaffold Image Credits - Palak Hirani

In order to accomplish successful tissue engineering, a scaffold's design should have a few essential components. To ensure optimal cell growth and survival, it should possess sufficient pore size and high porosity to facilitate effective waste, oxygen, and nutrition transfer as well as cell seeding and nutrient diffusion [17]. The scaffold should also have sufficient physical and mechanical strength, be biocompatible, and be able to integrate with surrounding tissues without the need for surgical removal. This will create an environment that is favourable for tissue regeneration and support, as seen in Figure 2 [18,19].

Functions

**Figure 3:**
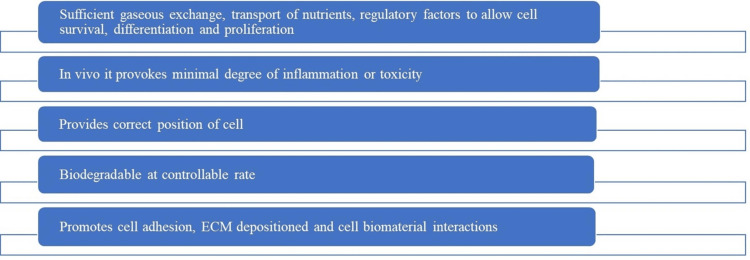
Function of Scaffold ECM- extracellular matrix Image Credits - Palak Hirani

Designed as three-dimensional porous solid biomaterials, scaffolds serve a variety of purposes such as promoting cell adhesion, promoting communication between the biomaterial and the cells and ensuring that extracellular matrix (ECM) is deposited where needed. Furthermore, as seen in Figure 3 [20], these scaffolds are critical in facilitating the effective transport of nutrients, which is necessary to sustain cell survival, proliferation, and differentiation and eventually aid in the success of tissue engineering and regeneration.

Classification

**Figure 4:**
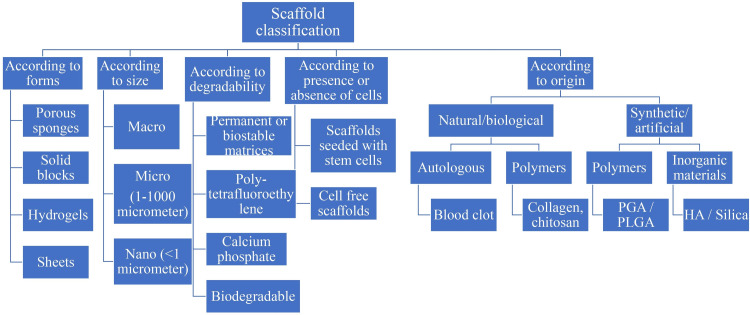
Examples of Scaffold PGA- polyglycolic acid PLGA- poly(lactic-co-glycolic acid) HA- hydroxyapatite Image Credits - Palak Hirani

As shown in Figure 4, scaffolds can be categorized into different categories according to their size, form, origin, degradability, and existence or devoid of cells [20].

Platelet-rich plasma

**Figure 5:**
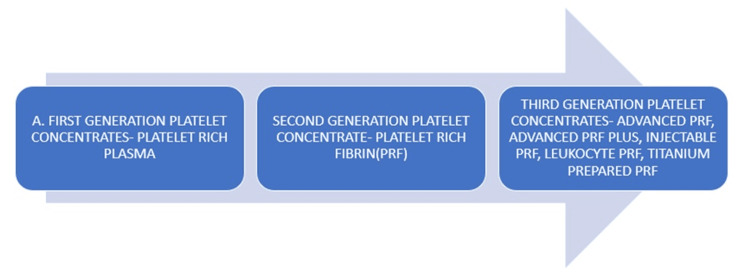
Platelet Rich Plasma Image Credits - Palak Hirani

When the blood of a patient is centrifuged, concentrates of platelets - products derived from blood - are obtained that comprise active platelets entwined inside a fibrin matrix scaffold. According to Figure 5, they are categorized [21,22].

A platelet concentrate’s second generation called platelet-rich fibrin (PRF) is utilized as a scaffold in tissue engineering for endodontics. PRF consists of a packed fibrin complex consisting of leukocytes, cytokines, and glycoproteins such as thrombospondin. In a condensed PRF scaffold, leukocytes hold an integral position in growth factor release in addition to an immune response. Promotion of tissue regeneration and wound healing is achieved due to this concentrated suspension of platelets rich in growth factors. Transforming growth factor beta (TGF-β) accelerates reactionary dentino-genesis by stimulating odontoblastic activity [23]. Leucocytes and autologous platelets combine to generate a complex fibrin matrix that aids in both soft and hard tissue repair [1]. PRF is classified into two groups based on the quantity of leukocytes: leukocyte-rich PRF, sometimes called advanced PRF (A-PRF), and pure PRF, also called leukocyte-poor PRF. The absence of chemical or biological additives means that PRF can avoid the negative reactions that come with them, which is one of its three main advantages. Secondly, because fibrin clots develop spontaneously, platelet activators like calcium chloride or bovine serum don't need to be added. Thirdly, the fibrin matrix collects a greater concentration of host immune cells.

Settlement of Platelet-Rich Plasma (PRP)

PRP is made in two steps: blood is drawn, and after an anticoagulant is added, it is centrifuged quickly. The top, bottom, and middle layers are where the buffy coat, erythrocytes, and plasma split off, respectively. Leukocytes and platelets are present in the buffy coat. After removing the blood cells, the remaining material is centrifuged more quickly. PRP is finally extracted from the lowest layer. Additions of 2% calcium chloride or bovine serum are used to produce insoluble PRP. This action stimulates the platelets and transforms plasmatic fibrinogen into insoluble fibrin scaffolds [24-26].

Low-speed centrifugation has become increasingly popular since it maintains more immune cells, GF, and cytokines, leading to the creation of A-PRF and injectable PRF (I-PRF). The conventional protocol involves centrifuging PRF for 12 minutes at 708 x g, A-PRF for eight minutes at 208 x g, and I-PRF for three minutes at 60 x g. I-PRF also has the benefit of producing a firm fibrin clot in 15 minutes, giving operators enough time to handle and mix with other materials [27].

PRF production should follow a specified procedure in order to obtain adequate amounts and quality of the fibrin matrix, leukocytes, platelets, and growth factors [28]. The method that is currently used to prepare PRF was described by Choukran et al. Butterfly needle of 24 gauge and 9 mL tubes to collect blood are present in the sample collection kit and are among the tools PRF preparation requires. A venous blood sample (5 ml) is drawn into 10 mL tubes without the use of an anticoagulant, and it is centrifuged right away for 10 minutes at 3000 rpm. Blood comes in proximity with the wall of the test tube during the process of centrifugation, activating platelets and starting the cascade of coagulation. Centrifugation is carried out right away for 10 minutes at 3000 rpm. Since there are no anticoagulants present, the majority of the platelets in the blood sample activate and start the coagulation cascade in a matter of minutes after coming into touch with the tube walls. A straw-coloured platelet-poor plasma layer is at the top of the final result, followed by a middle layer containing a PRF clot and red blood cells at the bottom. The circulating thrombin converts the fibrinogen into fibrin; the initial concentration is found at the upper portion within the tube. Consequently, a fibrin clot forms in the middle of the tube, with red blood cells at the top and acellular plasma at the bottom. Within the fibrin clot, the platelets are imprisoned [12]. Surgical tweezers are used to extract it from the test tube, and sterile scissors are employed to divide it off from the additional strata. Subsequently, the PRF is crushed between sterile gauze pads, forming an easily packed membranous layer within the root canals [13]. Rapid application of this method can aid in producing a PRF that is useful in clinical settings. The blood samples almost instantly start to coagulate when they come into touch with the glass tube if there is no anticoagulant present. If the blood collection and centrifugation processes take an excessive amount of time, this PRF preparation method may not succeed. Diffuse fibrin polymerization within the tube can result in the formation of a tiny, non-consistent blood clot [1]. PRF's role in regeneration of tissue and angiogenesis repair is a critical phase in the restoration of tissue health. An essential angiogenesis guide is PRF. The three-dimensional structure of fibrin gel and the activation of cytokines within meshes are responsible for the fibrin matrix angiogenesis property [1]. Table 1 shows various research pertaining to PRF.

**Table 1:** Summary Table HA- hydroxyapatite PRF- platelet-rich fibrin PRP- platelet-rich plasma ADMSC- adipose-derived mesenchymal stem cells MSC- mesenchymal stem cell RET- regenerative endodontic therapy CGF- concentrated growth factor REP- regenerative endodontic procedure CBCT- cone-beam computed tomography DPC- dystrophin-associated protein complex G-Rg1- ginsenoside Rg1 SNP- silver nanoparticles L-PRF- leucocyte and platelet-rich fibrin BMSC- bone marrow stem cells Image Credits - Palak Hirani

Name of Author	Year	Conclusion
Ohba S, et al [29]	2012	To enhance bone regeneration, electric polarization HA or PRP gel stimulates osteogenic cells.
Sun CK, et al [30]	2014	The left ventricle is protected by autologous ADMSC implanted in PRF.
Xu FT, et al [31]	2016	When G-Rg1 and PRF are combined, as opposed to when they are used separately, the effects of neovascularization and adipogenesis are enhanced.
Wang X, et al [32]	2017	In cases of significant bone deficiencies, the use of granular PRF and an MSC sheet mixed with nano HA enhances bone repair.
Zhang J, et al [33]	2017	Lyophilization maintains PRF's therapeutic benefits on tissue repair.
Raafat SN, et al [34]	2018	Compared to each substance, statins put on PRF stimulate superior bone development and repair.
Khorshidi H, et al [35]	2018	Higher mechanical strength and antibacterial qualities, particularly against viridans streptococci, are demonstrated by SNP modification of L-PRF.
Wang Z, et al [36]	2019	L PRF stimulates osteogenic differentiation, BMSC proliferation, and bone regeneration in mice by combining LPRF and BMSC.
Liu Z, et al [37]	2019	The combination of lyophilized and fresh PRF promotes bone formation in vivo and osteogenic differentiation in BMSC.
Steller D, et al [38]	2019	PRP and PRF help individuals with jaw osteonecrosis mend their bones more effectively.
ElSheshtawy AS, et al [39]	2020	When evaluating RET outcomes, standardized and calibrated two-dimensional radiography assessment proved to be just as successful as CBCT.
Kavitha M, et al [40]	2022	When autologous platelet concentrates such as PRF and CGF were used in REP, the results revealed root development, a reduction in the size of the apical foramen, and healing of the periapical lesion.
Li J, et al [41]	2023	When it came to the healing of periapical lesions, the elimination of clinical signs and symptoms, and the persistence of root development as scaffolds in RET, CGF and PRF performed similarly clinically.

PRP gel or electric polarization hydroxyapatite (HA) induces osteogenic cells to improve bone repair. The autologous adipose-derived mesenchymal stem cells (ADMSCs) implanted in PRF serve as protection for the left ventricle [29]. Combining ginsenoside Rg1 (G-Rg1) and PRF amplifies the effects of neovascularization and adipogenesis more so than using them independently [30]. Successful formation of the PRF human dental pulp cell (h-DPC) complex is achieved in the PRF preparation procedure by adding a dystrophin-associated protein complex (DPC) before blood centrifugation [31]. In cases of severe bone deficiency, the combination of granular PRF, a mesenchymal stem cell (MSC) sheet, and nano HA promotes better bone healing [32]. After lyophilization, PRF's therapeutic benefits for tissue healing are maintained [33]. Compared to other agents, statins on PRF promote superior bone growth and repair [34]. Silver nanoparticle (SNP) alteration of leucocyte and platelet-rich fibrin (L-PRF) demonstrates higher mechanical strength and antibacterial properties, especially against viridans streptococci [35]. L-PRF stimulates osteogenic differentiation, bone marrow stem cell (BMSC) proliferation, and bone regeneration in mice by combining L-PRF and BMSC [36]. Together, fresh and lyophilized PRF promotes bone formation in vivo and osteogenic differentiation in BMSC [37]. PRP and PRF facilitate more efficient bone mending in patients with jaw osteonecrosis [38]. When evaluating RET data, standardized and calibrated two-dimensional radiography assessment was performed just as well as cone-beam computed tomography (CBCT) [39]. The use of autologous platelet concentrates, such as PRF and concentrated growth factor (CGF), in regenerative endodontic procedures (REPs) resulted in root development, a reduction in the apical foramen's size, and periapical lesion repair. Regarding periapical lesion repair, clinical sign and symptom alleviation, and root growth persistence as scaffolds in RET, CGF, and PRF exhibited comparable clinical behavior [41].

Concentrates of leucocyte- and platelet-rich fibrin

Venous blood is extracted, placed in glass tubes, and centrifuged at less rpm. Without anticoagulants, platelet activation and fibrin polymerization occur instantly. There are numerous clinical uses for the PRF clot in the fields of oral, maxillofacial, ENT, and plastic surgery. One benefit of this preparation is that it dissolves gradually after application, and the three-dimensional fibrin mesh gradually reorganizes to resemble a blood clot in the body [41]. A second generation of platelet concentrates known as PRF - later dubbed leukocyte PRF, an improved form of PRP - was developed as a result of the preparation technique being optimized [3].

L-PRF, a blood derivative including cytokines, growth factors, and autologous platelets, leukocytes important for regeneration of tissue, is made by centrifuging the blood of patient. The processes of angiogenesis, chemotaxis, extracellular matrix formation, and cell proliferation and differentiation enable tissue regeneration. Considered the second generation of platelet concentrates, L-PRF is made up of structural glycoproteins, glucan chains, and a tightly knit mixture of cytokines encased in a fibrin network [1].

Advanced PRF

An upgraded version of PRF known as A-PRF has been identified, with a higher quantity of white blood cells. Leukocytes have been shown to be essential immune cells that can direct various cell types during the wound-healing process. There has been conjecture recently that lowering the centrifugal g-force can increase the amount of leukocytes in the PRF matrix because cell culture sinks toward the tubes for collection at the bottom due to severe centrifugation forces (700 rpm for 3 minutes). Since then, it has been observed that a decrease in centrifugal g-force increases the total count of leukocytes in the PRF matrix structures, which are currently referred to as advanced PRF or A-PRF. That being stated, this idea is supported by the fact that the A-PRF releases several growth factors which were demonstrated to be higher than in PRP and in L-PRF [42].

Injectable PRF 

The preparation of i-PRF was done using a horizontal centrifugation method for eight minutes at 200g. This results in a greater concentration of platelets and leukocytes (10.92 × 109 cells/L; 178% original values). Once the blood was collected, it was immediately placed in the centrifugation machine and run at 600 rpm and 44 × g for 8 minutes, following the low-speed centrifugation principle. Upon centrifugation, various blood components were generated in the bottom zone and yellow-orange-colored i-PRF in the upper zone [43].

Advanced (A) platelet-rich fibrin (PRF) plus (A-PRF+)

Further changes to the A-PRF technology resulted in the introduction of a novel formulation or preparation known as Advanced (A) -rich plasma plus (A-PRF+). Force of centrifugation directly affects the number of cells trapped inside the PRF matrix, researchers have worked to lower the centrifugal duration. Because of the total force, there is consequently cell loss. The development of the Fujioka-Kobayashi et al. [44] A-PRF+ preparation protocol was based on the results of a study conducted by Pavlovic et al. [43]. The protocol involved reducing the centrifugation speed to 1,300 rpm at 200 g and centrifuging for eight minutes [45].

Clinical Applications of A-PRF+

A-PRF+ is a successful treatment for intra-bony periodontal abnormalities and is a human autologous product having potential to enhance repair of periodontium. Significantly stronger and more viscoelastic membranes made from the A-PRF+ are indicative of viscoelasticity and superior strength in oral and periodontal procedures. With a greater potential, A-PRF+ is used as a supplement to the conventional regenerative treatment method for socket preservation [28]. A-PRF+ is a more potent and rapid regenerant in the treatment of alveolar osteitis, aiding in the advancements in pain management and soft and hard tissue healing. A-PRF+ usage is a safe, affordable technique to enhance postoperative quality in endodontic procedures [45].

Titanium (T) platelet-rich fibrin (T-PRF)

Titanium is non-corrosive and has good hemocompatibility. These are crucial features of the biomaterials that have interactions with blood. Platelets were stimulated in titanium and glass tubes in exactly the same way, and in the titanium tubes clot was developed which was observed to be clinically equal to those formed in the glass tubes. The fibrin structure of titanium-PRF seemed thicker and more tightly intertwined [3]. The fibrin carpet composed of titanium has a more robust network structure. A strong fibrin structure is necessary to increase the amount of time needed for fibrin resorption and growth factor release. T-PRF is also used to prevent silica contamination problems and any detrimental effects that dry tubes containing glass may have in the short and long term [27].

T-PRF Preparation

The patient's antecubital vein was used to extract a 10-milliliter blood sample, which was then immediately placed in a grade IV titanium tube and centrifuged for 10 minutes at 3000 rpm at room temperature. After being liberated from RBCs, the resulting T-PRF clot was placed on sterile gauze and allowed serum to seep for 20 minutes. Growth factors such as vascular endothelial growth factor (VEGF), transforming growth factor (TGF), and platelet derived growth factor (PDGF) have been shown to be released at greater levels in T-PRF [42].

Clinical Applications of T-PRF

Mitra et al.’s study discusses periodontal procedures like osseous defects and furcation disorders. A study conducted by Olgun et al. examined the T-PRF’s utility in sinus lift procedures. The accepted gold standard for root coverage, connective tissue grafts (CTGs), can be replaced with T-PRF when used as autogenous material. Clinical metrics improved with T-PRF, indicating that they might aid in the soft and hard healing of tissue in intra-bony lesions [5]. T-PRF membranes in conjunction with open flap debridement (OFD) results in a considerable release of more concentrations of growth factor and in GCF decreased ratio of "RANKL/OPG". T-PRF aids in the treatment of endo-perio lesions by lowering pocket depth and increasing attachment level [46].

Newer advances

Procedures such as leukocyte-rich PRF, which are affordable, straightforward, and effective, will find widespread application in implant dentistry in the future. PRF and gel-formulated PRP are novel ideas that need further study and testing in regenerative dentistry because they have a lot of clinical potential in periodontal and dentoalveolar procedures [28].

PRF-Lysate (Ly) (PRF-Ly)

PRF lysate, a novel PRF derivative, involves collecting and incubating PRF exudate at 37°C with 5% CO2. It contains VEGF, TGF, PDGF, and EGF as growth factors, believed to aid healing from chronic UV radiation exposure by enhancing fibroblast proliferation, migration, and collagen deposition. Further research will evaluate its efficacy in this innovative application [47].

Lyophilized (Ly)-PRF (Ly-PRF)

For the preparation of lyophilized PRF, PRF membranes were frozen and stored at −80 °C. The frozen PRF was then freeze dried overnight using a Labconco lyophilizer at −51 °C (Free Zone, Labconco, Kansas City, MO, USA).

Fabrication: Ly-PRF fabrication method was recommended by Ngah et al. (2018) [47], building upon techniques by Kardos et al. (2018) [48] and Li et al. (2014) [49]. This choice stems from Ly-PRF's proven effectiveness in both lab and animal trials for cranial bone repair. The publication of this Ly-PRF creation process first time in scientific literature, ensuring consistency by following similar methodologies [42]. The produced Ly-PRF shares properties akin to a sponge, offering an accessible and natural means to generate a platelet concentrate releasing growth factors continuously. Its versatility extends to serving as a viable biomaterial for craniofacial scaffolding, showcasing not only its role as a growth factor repository but also fundamental scaffold attributes [47].

Albumin (Alb) PRF (Alb-PRF)

This by-product of blood is created through two procedures that follow centrifugation: heating and cell incorporation (heated serum, low platelet plasma). It is made completely of homologous blood, in which the PRF and factors affecting growth or growth factor cytokines have been extracted and made liquid from the red blood cell junction or leukocyte zone [5]. In vitro testing and translational studies on this newest biomaterial have already advanced in this time period. It is believed that Alb-PRF will provide excellent outcomes in the fields of oral/periodontal, cosmetic, and face medicine [50].

## Conclusions

In conclusion, scaffolds are integral to tissue engineering, offering crucial support for cell adhesion, extracellular matrix deposition, and effective nutrient transport. PRP and PRF have emerged as valuable tools in regenerative medicine, with PRF exhibiting particular promise due to its role in angiogenesis and its diverse clinical applications. Different forms of PRF, such as A-PRF and I-PRF, have improved leukocyte and growth factor concentrations, enhancing their potential for tissue regeneration. Research studies have demonstrated their effectiveness in various medical fields, including oral and maxillofacial surgery. The continuous evolution of platelet concentrates like PRF holds great potential for advancing the field of tissue regeneration and repair.
